# Correction: Recent advances in the methanol synthesis *via* methane reforming processes

**DOI:** 10.1039/d0ra90096f

**Published:** 2020-09-14

**Authors:** Muhammad Usman Rashid, W. M. A. Wan Daud

**Affiliations:** a Department of Chemical Engineering, University of Malaya 50603 Kuala Lumpur Malaysia ashri@um.edu.my +60 379675319 +60 379675297

## Abstract

Correction for ‘Recent advances in the methanol synthesis *via* methane reforming processes’ by Muhammad Usman Rashid *et al.*, *RSC Adv.*, 2015, **5**, 21945–21972, DOI: 10.1039/C4RA15625K.

The authors regret that the name of one of the authors (Muhammad Usman Rashid) was shown incorrectly in the original article. The corrected author list is as shown above.

The Royal Society of Chemistry apologises for these errors and any consequent inconvenience to authors and readers.

## Supplementary Material

